# Supplementary material for: Ghost-arc geochemical anomaly at a spreading ridge caused by supersized flat subduction
Source: Nat Commun. 2023 Apr 12;14:2083. doi: 10.1038/s41467-023-37799-w (PMC10097660; doi:10.1038/s41467-023-37799-w)
Supplement: Supplementary file 1 — Supplementary information [file 41467_2023_37799_MOESM1_ESM.pdf]

Supplementary Data for:
Ghost-arc geochemical anomaly at a spreading ridge caused by supersized flat subduction

Guido M. Gianni[1,2], Jeremías Likerman[2,3], César R. Navarrete[2,4†], Conrado R. Gianni[1†] and Sergio Zlotnik[5,6*†]

[1]Instituto Geofísico Sismológico Ing. Fernando Volponi (IGSV), Universidad Nacional de San Juan, San Juan, Argentina.
[2]National Scientific and Technical Research Council (CONICET), Capital Federal, Argentina.
[3]Instituto de Estudios Andinos Don Pablo Groeber, Universidad de Buenos Aires, Capital Federal, Argentina.
[4]Laboratorio Patagónico de Petro-Tectónica, Universidad Nacional de la Patagonia "San Juan Bosco", Comodoro Rivadavia, Chubut, Argentina.
[5*]Laboratori de Càlcul Numèric, Escola Tècnica Superior d'Enginyers de Camins, Canals i Ports, Universitat Politècnica de Catalunya, Barcelona, Spain.
[6*]Centre Internacional de Mètodes Numèrics a l'Enginyeria (CIMNE), Barcelona, Spain.

*Corresponding author(s). E-mail(s): sergio.zlotnik@upc.edu; Contributing authors: guidogianni22@gmail.com; jlikerman@gl.fcen.uba.ar; cesarnavarrete@live.com.ar; io_g@hotmail.com;
†These authors contributed equally to this work.

Extended Data

This PDF file includes:
Supplementary Figs. 1 to 6
Supplementary Tables 1 to 3
Supplementary Data file 1, 2, and 3 (Separate files)

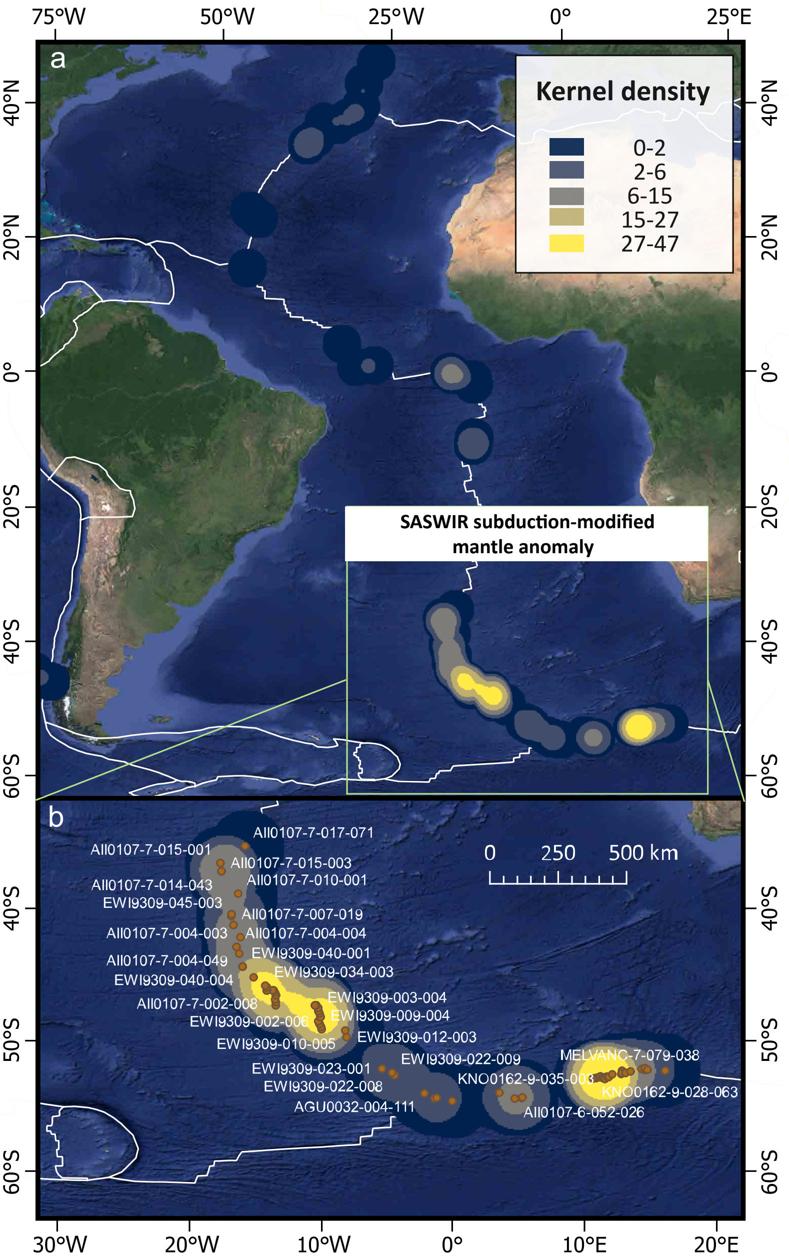

Fig. 1 Kernel density map of MORBs with ghost-arc geochemical signatures along the Central and Southern Atlantic MOR..a, Kernel density map build with data from Yang et al.[19], showing the localized and accentuated character of the SASWIR subduction-modified mantle anomaly. **b**, Close-up view depicting MORB samples in the study area forming a continuous belt of ghost-arc geochemical signatures along aprox. 4,400 km. Geochemical MORB compilation is found in Supplementary Data file 1.

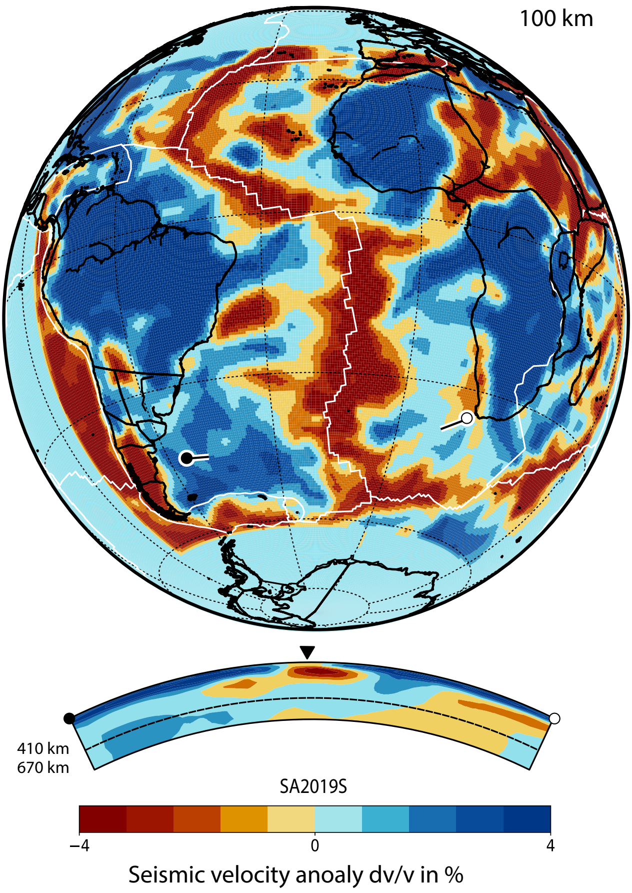

Fig. 2 Seismic tomography slice from the regional S-wave SA2019S model[30]. The dv/v -1 and -2 % low-velocity anomalies were used as a reference to map the active mantle source beneath the SASWIR.

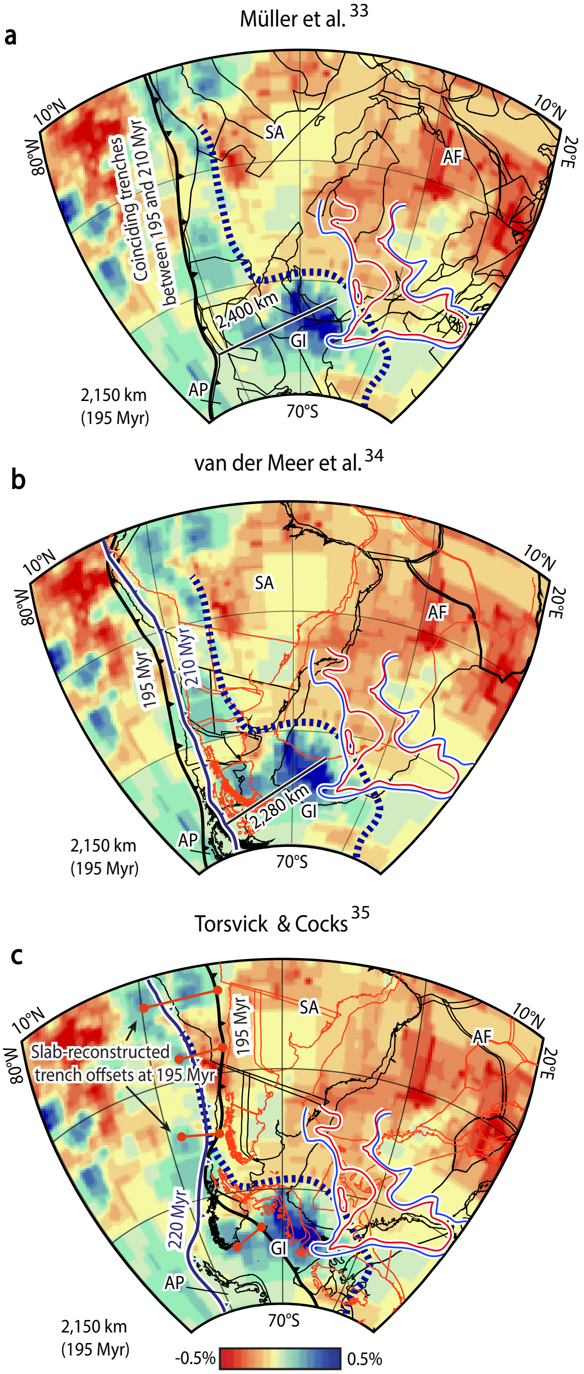

Fig. 3 Plate kinematic reconstructions and mantle analysis of the Southwestern Gondwana margin implementing alternative reference frames. Plate reconstruction implementing the **a**, lower mantle slab reference frame of van der Meer et al.[33], **b**, the tectonic rules-based mantle reference frame of Müller et al.[34], and **c** the mantle reference frame of Torsvik and Cocks[35] considering the plume generation zone approach. Abbreviations are; SA: South American plate, AF: African plate, and GI: Georgia Islands slab.

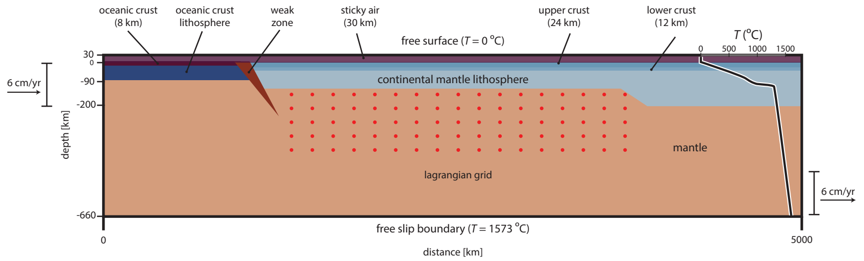

Fig. 4 2-D Numerical model set up. Image illustrating model geometry, parameters, and boundary conditions. This figure also depicts the locations of the Lagrangian markers used to track mantle flow paths during flat subduction development. A weak detachment-like rheological anomaly is introduced to localize deformation and induce subduction.

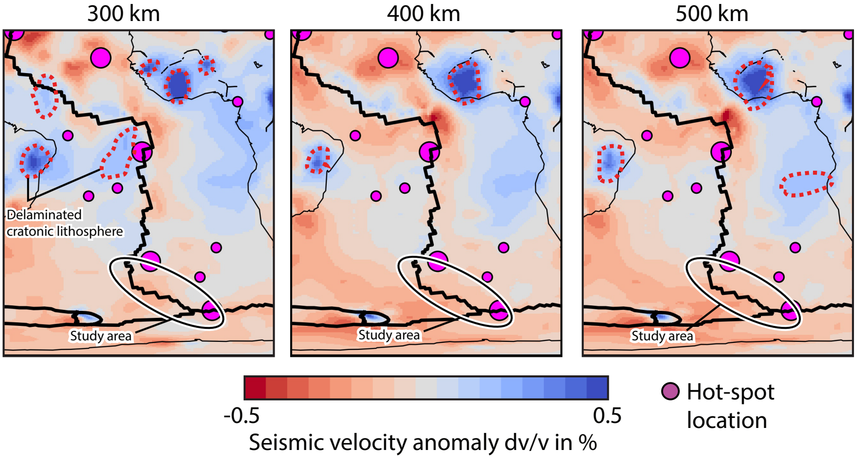

Fig. 5 Seismic tomography slices at different upper mantle depths from the MITP08 global seismic tomography model[78]. This model was used by Hu et al.[53] to detect neutrally buoyant cratonic lithospheric blocks delaminated beneath the South Atlantic MORB since the Cretaceous. This analysis of tomographic slices in the upper mantle of the South Atlantic and Southwest Indian oceans show a lack of visible delaminated cratonic lithosphere beneath the study area.

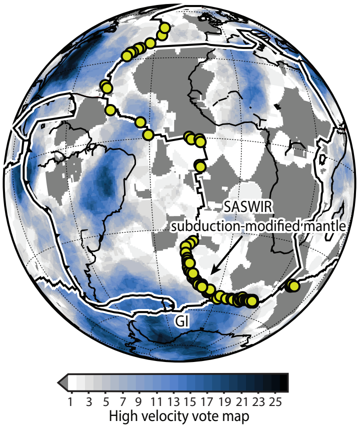

Fig. 6 High-velocity vote map at 2,100 km with samples with ghost-arc geochemical signatures. This image illustrates the spatial relationship between Triassic slab records and localized samples with ghost-arc geochemical signatures scattered along the Atlantic-Southwest Indian MOR from Yang et al.[19].

Table 1 Global P- and S-wave tomography models used to construct the tomographic vote maps shown in Fig. 3c and Supplementary Fig. 6.

**Global seismic tomography models**					
**Model**	**Data type**	**Reference model**	**Model**	**Data type**	**Reference model**
GyPSuM-S[69]	Body waves	TNA/SNA	MITP08[78]	Body waves	AK135
DETOX-P2[70]	Body waves (P and Pdiff)	IASP91	UU-P07[36]	Body waves (P, PP and Pdiff)	AK135
DETOX-P3[70]	Body waves (P, PP and Pdiff)	IASP91	TX2019Slab-P[79]	Body waves	AK135
HMSL-P06[71]	Surface waves, body waves	AK135 for ray tracing, traveltime measurements for each phase have the mean removed	TX2019Slab-S[79]	Body waves	TNA/SNA
HMSL-S06[71]	Surface waves, body waves	AK135 for ray tracing, traveltime measurements for each phase have the mean removed	S362ANI+M[80]	Surface waves, body waves, normal modes	STW105
PRI-P05[72]	Body waves	IASP91	S20RTS[81]	Surface waves, body waves, normal modes	REM
PRI-S05[72]	Body waves	IASP91	S40RTS[82]	Surface waves, body waves, normal modes	REM
SPani-P[73]	Surface waves, body waves	PREM	SAVANI[83]	Surface waves, body waves	ALA
SPani-S[73]	Surface waves, body waves	PREM	SAW642ANb[84]	Waveform	REM
GAP-P4[74]	Body waves	GAP	SEMUCB-WM[85]	Waveform	Custom averaged model
LLNL_G3Dv3[75]	Body waves	Custom averaged model	SEMum[86]	Waveform	PREM
Hosseini2016[76]	Body waves	IASP91	TX2011[87]	Body waves	TX2011ref
SEISGLOB1[77]	Surface waves, normal modes	PREM	TX2015[88]	Body waves	TX2011ref

Table 2 Model parameters of partial melting

Latent heat of fusion (kJ/kg)	250
Solidus coefficient ‘as’ (k)	1000
Solidus coefficient ‘bs’ (k/Pa)	-1.2e^{-7}
Solidus coefficient ‘cs’ (k/Pa2)	0.5e^{-16}
Liquidus coefficient ‘al’ (k)	1493
Liquidus coefficient ‘bl’ (k/Pa)	-1.2e^{-7}
Liquidus coefficient ‘cl’ (k/Pa2)	1.6e^{-16}
Viscous softening melt fraction	0.15-0.30

Table 3 Material Properties and model parameters

	Oceanic crust	Oceanic crust lithosphere	Continental upper crust	Continental lower crust	Continental mantle lithosphere	Mantle	
Viscous rheology							
Material	Wet Olivine	Wet olivine	Wet quartzite	Dry Maryland diabase	Wet olivine	Dry olivine Diffusion	Dry olivine Dislocation
Aps (Pa^{-n} s^{-1})	5.04×10^{-28}	1.76×10^{-14}	8.57×10^{-28}	5.04×10^{-28}	1.76×10^{-14}	1.5	1.1
n	4.7	3	4	4.7	3	3.5	1
E (kJ mol^{-1})	484	430	223	485	430	520	375
V* (cm^3 mol^{-1})	0	10	0	0	10	6	2.3
Density							
ρ (kg m^{-3})	2950	3200	2800	2950	3200	3250	
α (K^{-1})	$3.0x10^{-5}$	$3.0x10^{-5}$	$3.0x10^{-5}$	$3.0x10^{-5}$	$3.0x10^{-5}$	3.0x10-5	
Plastic rheology							
Co (MPa)	10	10	20	20	20	10	
Cohesion after softening (MPa)	2	2	3	3	3	10	
Friction coefficient	0.577	0.577	0.44	0.44	0.44	0.6	
Friction coefficient after softening	0.1154	0.1154	0.088	0.088	0.088	0.6	
Strain range of softening	0.0-2.0	0.0-2.0	0.0-0.5	0.0-0.5	0.0-0.5	1.5	
