# Supplementary material for: Ghost-arc geochemical anomaly at a spreading ridge caused by supersized flat subduction
Source: Nat Commun. 2023 Apr 12;14:2083. doi: 10.1038/s41467-023-37799-w (PMC10097660; doi:10.1038/s41467-023-37799-w)
Supplement: Supplementary file 3 — Description of Additional Supplementary files [file 41467_2023_37799_MOESM3_ESM.pdf]

Description of Additional Supplementary Files

File name: Supplementary Data 1
Description: Compilation of Global location of MORBs with backarc basin basaltlike geochemical signatures19used in Supplementary Fig. 1 to build the Kernel density map (separate file).

File name: Supplementary Data 2
Description: Compiled geochemical data of MORBs from the Pacific MOR and SASWIR19 used in Fig. 2 (separate file).

File name: Supplementary Data 3
Description: Compiled geochronological data of Paleozoic to Mesozoic igneous rocks42 along the Southwest margin of Gondwana used in Fig. 4 (separate file).
